# Association between Neck Circumference and the Occurrence of Cardiovascular Events in Type 2 Diabetes: Beijing Community Diabetes Study 20 (BCDS-20)

**DOI:** 10.1155/2019/4242304

**Published:** 2019-11-11

**Authors:** Guang-Ran Yang, Ming-Xia Yuan, Gang Wan, Xue-Lian Zhang, Han-Jing Fu, Shen-Yuan Yuan, Liang-Xiang Zhu, Rong-Rong Xie, Jian-Dong Zhang, Yu-Ling Li, Yan-Hua Sun, Qin-Fang Dai, Da-Yong Gao, Xue-Li Cui, Jian-Qin Gao, Zi-Ming Wang, Ying-Jun Chen, Dong-Ming Hu, Juan Gao, Liyong Bai

**Affiliations:** ^1^Department of Endocrinology, Beijing Tongren Hospital, Capital Medical University, Beijing, China; ^2^Department of Medical Records and Statistics, Beijing Ditan Hospital, Capital Medical University, Beijing, China; ^3^Jinsong Community Health Service Center, Beijing, China; ^4^Xinjiekou Community Health Service Center, Beijing, China; ^5^Cuigezhuang Community Health Service Center, Beijing, China; ^6^Yuetan Community Health Service Center of Fuxing Hospital, Capital Medical University, Beijing, China; ^7^Aerospace Central Hospital, Beijing, China; ^8^Sanlitun Community Health Service Center, Beijing, China; ^9^Department of Endocrinology, Beijing Aerospace General Hospital, Beijing, China; ^10^Jiangtai Community Health Service Center, Beijing, China; ^11^Majiapu Community Health Service Center, Beijing, China; ^12^Zuojiazhuang Community Health Service Center, Beijing, China; ^13^Balizhuang Community Health Service Center, Beijing, China; ^14^Bayer Healthcare Company Limited, Beijing, China

## Abstract

**Background:**

Neck circumference (NC) was found to be related to the risk factors of cardiovascular disease. However, the effects of NC on cardiovascular disease are still controversial. A prospective study of Chinese patients with type 2 diabetes was performed to evaluate the relationship between NC and cardiovascular disease.

**Methods:**

A multicenter prospective study with eight-year follow-up was conducted in Beijing communities. Cardiovascular events were defined as myocardial infarction, unstable angina pectoris, hospitalization for heart failure, coronary revascularization, cardiac death, stroke, transient ischemic attack, and cerebral hemorrhage.

**Results:**

A total of 3,009 diabetic patients were recruited. Following an eight-year follow-up, 211 patients with cardiovascular events (105 in men and 106 in women) were identified. All patients were categorized into two groups according to the upper quartile of NC (43 cm in men and 39 cm in women). The prevalence of cardiovascular events in men with an NC >43 cm (16.48%) was higher than that in the group with an NC <43 cm (8.16%, *p*=0.007). The prevalence of cardiovascular events in women with the NC >39 cm (10.67%) was higher compared to the group with NC <39 cm (5.31%, *p*=0.004). The longitudinal prevalence of cardiovascular events in groups with different NC increased with the increasing duration of follow-up (*p* < 0.001). Cox regression analysis showed that higher NC was associated with the occurrence of cardiovascular events after adjusting for confounding variables (adjusted HR = 2.305 (1.535–3.460)).

**Conclusions:**

NC was associated with the occurrence of cardiovascular events in type 2 diabetes in Chinese communities, and greater NC may increase the risk of cardiovascular events by about 2.3-fold.

## 1. Introduction

Cardiovascular disease (CVD) is the leading cause of death among people with type 2 diabetes in China. It was reported that CVD risk is 2- to 8-fold higher in the diabetic population than in nondiabetic patients of a similar age, sex, and ethnicity [1, 2]. Type 2 diabetes is associated with many risk factors for CVD, with a prevalence of 75% to 85% for hypertension, 70% to 80% for dyslipidemia, and 60% to 70% for obesity among adults with diabetes [3–5].

Overweight/obesity has been shown to be an important risk factor for CVD. In clinical practice, BMI, waist circumference (WC), and waist-to-hip ratio are used to assess overweight/obesity.

Neck circumference (NC), an index for upper-body subcutaneous adipose distribution, was proved to be independently correlated with abdominal obesity [6]. The association between NC and insulin resistance [7–9], metabolic syndrome [10–12], and early-stage atherosclerosis [13] has been studied. However, the ability of NC to have any value in predicting CVD is controversial [14–16]. In the Framingham heart study, NC was not related to the incidence of cardiovascular events even after adjusting multiple variables [14]. However, in another prospective cohort study, a higher NC was related to a higher incidence of future CVD events and all-cause mortality in high-risk cardiology outpatients [15]. A cross-sectional study performed on patients with coronary artery disease who underwent coronary angiography showed that NC was much better in predicting the risk of coronary artery disease than waist-to-height ratio, waist-to-hip ratio, and BMI [16].

There is a lack of prospective studies on whether NC could predict future CVD events in Chinese patients with type 2 diabetes. The aim of this study (the Beijing Community Diabetes Study (BCDS) 20) was to evaluate the association between NC and the occurrence of CVD events in patients with type 2 diabetes following an eight-year management in Beijing communities.

## 2. Research Design and Methods

### 2.1. Participants

This was a prospective, multicenter, eight-year-long study in Beijing communities. A multistage random sampling method was performed to select the community health centers in Beijing. Type 2 diabetes patients aged 20–80 years who had lived in the same community for more than 5 years were enrolled in this study between August 2008 and July 2009 [17]. There were 3,009 diabetic patients from thirteen community health centers who were recruited. The thirteen community health centers included Cuigezhuang, Jinsong, Xinjiekou, Yuetan, Donggaodi, Mingzu, Yongdinglu, Sanlitun, Jiangtai, Shazikou, Balizhuang, Zuojiazhuang, and Majiapu [17]. People with severe disabilities, hepatic failure, renal failure, schizophrenia, or goiter were not included [17]. After the eight-year management, 25 patients died from cardiac events.

The Ethics Committee of Beijing Tongren Hospital, Capital Medical University, reviewed and approved this study. This study was conducted in accordance with the provisions of the Declaration of Helsinki. All participants gave informed consent [17].

### 2.2. Integrated Care

Details of the design, methods, population, and main components of integrated care have been published previously [17]. Management targets were defined in accordance with the *Chinese Guideline for Type 2 Diabetes* [18]: (1) HbA1c < 7%, (2) fasting plasma glucose (FPG) < 7.2 mmol/l, (3) BP < 130/80 mmHg, and (4) LDL-C < 2.6 mmol/l.

All the type 2 diabetes patients were managed by general practitioners in the community health centers, and specialists from Beijing Tongren hospital were consulted. According to the protocol, at baseline and each follow-up visits, a physical examination and laboratory measurements were performed. BMI was calculated as weight divided by height squared (kg/m^2^). WC was measured at the level midway between the lower rib margin and the iliac crest. NC was measured with people's head erect and eye facing forward, horizontally at the upper margin of the laryngeal prominence (Adam's apple) [17, 19].

FPG and HbA1c were measured four times, and the lipid profiles two times every year. FPG and lipid profiles were measured by using an autoanalyzer. A Bio-Rad Variant hemoglobin analyzer was used to measure HbA1c [17].

The primary endpoints were the occurrence of CVD events including myocardial infarction, unstable angina pectoris, hospitalization for heart failure, coronary revascularization, cardiac death, stroke (as confirmed by computed tomography/magnetic resonance imaging brain scan), transient ischemic attack, and cerebral hemorrhage. CVD events were confirmed by reviewing hospital records and were classified by an endpoint committee comprised of cardiologists, neurologists, and endocrinologists.

### 2.3. Statistical Analysis

The database was established using EpiData 3.0 software. SAS software (SAS Institute Inc., Cary, NC) was used in data analysis. All results were expressed as mean (±SD), *n* (percent), or median (range). The significance of differences between continuous variables was assessed by a *t*-test. However, because the duration of diabetes was not normally distributed, the rank sum test was used. Kaplan–Meier analysis was used to assess the cumulative percent of CVD events between different NC groups by follow-up time, and then the log-rank test was used to assess the difference between the two NC groups. Cox regression analysis was utilized to estimate the hazard ratio (HR) and 95% CI for the effects of NC on CVD risk. A *p* value <0.05 in 2-tailed tests was considered to be statistically significant.

## 3. Results

### 3.1. Demographic Characteristics

At baseline, 3,009 people were enrolled (1194 men and 1815 women). The mean age was 67.62 ± 10.69 years, with NC of 36.43 ± 3.84 cm. The median duration of diabetes was 10.1 years. The mean HbA1c was 7.35 ± 1.59%. There were statistical differences in NC, WC, diastolic blood pressure (DBP), total cholesterol (TC), HDL, LDL, and smoking between men and women (Table 1).

### 3.2. The Prevalence of CVD Events and Its Relationship with NC

After eight-year management, 211 CVD events occurred (105 in men and 106 in women). All the patients were categorized into two groups by gender and then further categorized into two subgroups according to having CVD events or not. In men, patients having CVD events were much older, with longer duration of diabetes and higher systolic blood pressure (SBP) and WC (*p* < 0.05, Table 2). More people having CVD events took antihypertension medication (68.57%) and antihyglycemic medication (97.14%, *p* < 0.05, Table 2). However, there was no statistical significance in BMI, NC, FPG, HbA1c, and lipid profile (Table 2). In the female group, there were significant statistical differences in age, NC, SBP, and the percentage of smoking between women with CVD events and without CVD events (*p* < 0.05, Table 3). More women took antihypertension medication (75.47%) in the CVD group (*p* < 0.05, Table 3). However, there was no statistical significance in the use of hyperglycemic medication and statin between women with CVD events and without CVD events (*p* > 0.05, Table 3). Compared to the baseline data, NC did not change in the CVD and without CVD groups in men and women after eight-year follow-up (38.22 ± 2.74 cm and 38.16 ± 3.53 cm in men; 35.83 ± 2.67 cm and 35.13 ± 3.06 cm in women, respectively, all *p* < 0.05).

### 3.3. The Incidence of CVD Events in NC Subgroups

**Figure 1:**
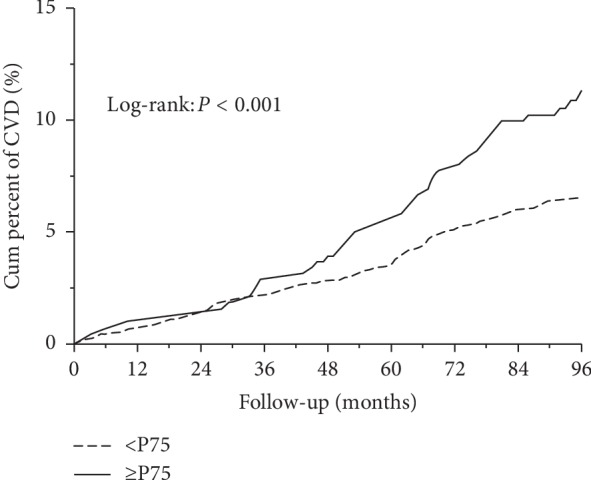
Kaplan–Meier estimates for neck circumference with cardiovascular events. <P75: neck circumference < the upper quartile group and ≥P75: neck circumference ≥ the upper quartile group.

The 25th, 50th, and 75th quartiles of NC in men and women were calculated. The 25th, 50th, and 75th quartiles of NC were 40 cm, 41 cm, and 43 cm in men and 36 cm, 38 cm, and 39 cm in women. High NC was defined when NC was above the 75th quartile. The prevalence of CVD in the NC >43 cm group in men (16.48%) was statistically significant when compared with the group that had NC <43 cm (8.16%, *p*=0.007). Similar results were observed in women. The prevalence of CVD in the NC >39 cm group (10.67%) was higher than that in the group with NC <39 cm (5.31%, *p*=0.004). The longitudinal prevalence of CVD events in different NC groups increased with the follow-up (log-rank test, *χ*^2^ = 14.81, *p* < 0.001, Figure 1).

### 3.4. Cox Regression Analysis between NC and Risk Factors for CVD

The Cox regression model was utilized to evaluate the effects of NC on CVD events. The upper quartile of NC was set as high NC. High NC was found to be associated with the occurrence of CVD (crude HR = 2.126 (95% CI 1.231–3.673) in men and 2.043 (95% CI 1.244–3.356) in women).

The adjusted HR for CVD was 2.051 (95% CI 1.420–2.962, *p* < 0.001) in the age-, smoking-, and sex-adjusted model. After further adjusting for SBP, HbA1c, LDL, diabetic duration, and education attainments and the use of antihypertension medication, antiglycemic medication, and statin, this association persisted; adjusted HR = 2.305 (1.535–3.460, *p* < 0.001, Table 4).

## 4. Discussion

In this prospective multicenter study, the prevalence of CVD events increased with NC enlargement. Moreover, Cox regression showed that larger NC may increase the occurrence of CVD events by about 2.3-fold in type 2 diabetes in Chinese patients after adjusting for age, gender, smoking, SBP, LDL, HbA1c, duration of diabetes, education attainments, and medication.

There are several anthropometric indicators to evaluate overweight/obesity, such as BMI, WC, waist-hip ratio, and NC. Initially, BMI was used to evaluate fat distribution in diabetes in clinical practice. Later, waist-hip ratio and WC were suggested to assess abdominal obesity in diabetes. However, it is not always practical to use these indicators, especially in winter. Additionally, the measurement of WC differs between the preprandial and postprandial periods especially in obese people. NC was first evaluated in relation to cardiovascular risk factors by Sjöström et al. in 1995 [20], and many studies were performed to assess the effects of NC in clinical practice. NC was initially used to evaluate overweight and obesity [8, 10, 21]. However, whether NC could be an anthropometric indicator in evaluating obesity in type 2 diabetes was not well known ten years ago. In 2008, NC measurement was included in the physical examination in this prospective, multicenter study. In our previous analysis, NC was found to be positively correlated to overweight/obesity in type 2 diabetes [19]. Since then, NC has been used as an indicator for evaluating overweight/obesity in many studies. In other studies, it was used to assess obesity-related diseases, such as insulin resistance, obstructive sleep apnea syndrome, and metabolic syndrome [9, 11].

Consistent with our findings, NC was proved to be related to cardiovascular risk factors (lipid profile, insulin resistance, metabolic syndrome, and hypertension) in some studies [9, 11, 19, 21]. The association between NC and CVD risk factors was confirmed in many studies [7, 12, 22].

Therefore, NC was presumed to be related to CVD. In a cross-sectional study, NC was found to be better in predicting the risk of coronary artery disease than other anthropometric indices in patients with stable angina who underwent angiography [16]. Another cross-sectional study performed on patients undergoing elective coronary angiography also showed that NC was correlated with coronary angiographic severity scoring [23]. However, the results from the ELSA-Brazil study found that NC was not associated with coronary atherosclerosis [24].

Prospective studies are needed to determine whether NC can predict future CVD events. In a Chinese cohort study conducted in a high-risk population, NC was found to be related to the incidence of CHD events after a mean 8.8-year follow-up [15]. In our study of type 2 diabetes patients, higher NC was associated with the occurrence of CVD events after an eight-year follow-up. However, in the Framingham study, though NC was related to the CVD risk factor, NC was not related to the incidence of CVD events after a mean of 6.7 years of follow-up [14].

Differences in the patient population and the definition of CVD events might be the other reasons for this discrepancy. Type 2 diabetes patients always have hypertension and dyslipidemia, which are all risk factors for CVD. They have a higher risk for CVD events than the general population. However, in this study, the association between higher NC and incidence of CVD events persisted even after adjusting for other CVD risk factors, such as BP, LDL, HbA1c, and duration of diabetes. In the cohort study which found that NC was related to the incidence of CHD events, patients with high-risk for CVD were recruited [15]. It seemed that NC might be associated with CVD events in high-risk patients but not in the general population. Another reason might be related to age, which is an important risk factor for CVD. The mean age in this study is 67 years. This is older than that in the Framingham study [14]. Third, the follow-up period was eight years in this study. In the cohort study conducted in the Chinese population with high risk for CVD, the follow-up period was 8.8 years [15]. However, in the Framingham study, it was 6.7 years [14]. Different follow-up periods may be another reason for this discrepancy. As shown in this study, with the longer duration of follow-up, the occurrence of CVD events in the higher NC group increased dramatically. Therefore, to predict CVD risk using NC, it might take at least eight years for high-risk patients and more than eight years for the general population.

This study has its limitations. First, all the type 2 diabetes patients were managed according to the Chinese guideline for type 2 diabetes. The management might affect the results even though the mean HbA1c and LDL did not meet the control target after eight-year management. Second, in this study, we enrolled only patients with type 2 diabetes. Type 2 diabetes patients have a higher risk for CVD than the general population [1, 2]. Further large-scale studies are needed to confirm the relationship between NC and CVD risk.

In conclusion, this study indicated that larger NC may be associated with the occurrence of CVD events in Chinese patients with type 2 diabetes. Higher NC might be an independent risk factor for predicting CVD events in type 2 diabetes. Given the high incidence of CVD events in type 2 diabetes, further large-scale and prospective studies in the general population may be worthwhile to confirm this relationship between NC and CVD risk.

## Figures and Tables

**Table 1:** Baseline clinical characteristics in men and women.

	Total (*n*=3009)	Men (*n*=1194)	Women (*n*=1815)	*t* value	*p* value
Age (years)	67.62 ± 10.69	67.22 ± 12.11	67.88 ± 9.64	−1.59	0.113
BMI (kg/m^2^)	26.20 ± 44.54	25.30 ± 3.51	26.79 ± 57.28	−1.11	0.267
Diabetic duration (years)	10.1 (6.5, 15.4)	10.1 (6.1, 15.3)	10.1 (6.8, 15.4)	0.58^*∗*^ (*Z*)	0.565
Smoking (*n*, %)	479 (15.93)	386 (32.33)	93 (5.13)	397.67^#^ (x2)	<0.001
WC (cm)	89.06 ± 9.46	91.10 ± 8.81	87.72 ± 9.64	9.91	<0.001
NC (cm)	36.43 ± 3.84	38.20 ± 3.80	35.26 ± 3.40	21.62	<0.001
SBP (mmHg)	129.46 ± 14.60	129.53 ± 14.50	129.41 ± 14.67	0.21	0.832
DBP (mmHg)	77.57 ± 8.99	78.54 ± 9.06	76.94 ± 8.90	4.75	<0.001
FPG (mmol/L)	7.83 ± 2.57	7.90 ± 2.69	7.78 ± 2.48	1.22	0.222
HbA1c (%)	7.35 ± 1.59	7.39 ± 1.67	7.32 ± 1.54	1.23	0.219
TG (mmol/L)	1.85 ± 1.34	1.80 ± 1.48	1.88 ± 1.24	−1.60	0.109
TC (mmol/L)	5.22 ± 1.23	5.03 ± 1.20	5.35 ± 1.24	−6.80	<0.001
HDL (mmol/L)	1.32 ± 0.47	1.24 ± 0.41	1.38 ± 0.50	−7.98	<0.001
LDL (mmol/L)	3.07 ± 0.93	2.94 ± 0.88	3.15 ± 0.95	−5.91	<0.001
Antihypertension medication (*n*, %)	1805 (59.99)	669 (56.03)	1136 (62.59)	12.91^#^ (x2)	<0.001
Antiglycemic medication (*n*, %)	2733 (90.83)	1073 (89.87)	1660 (91.46)	2.20^#^ (x2)	0.138
Statin (*n*, %)	1948 (64.74)	730 (61.14)	1218 (67.11)	11.24^#^ (x2)	0.001

^*∗*^Rank sum test was used, expressed as median (range). ^#^Chi-square was used. BMI, body mass index; WC, waist circumference; NC, neck circumference; SBP, systolic blood pressure; DBP, diastolic blood pressure; FPG, fasting plasma glucose; HbA1c, hemoglobin A1c; TG, triglyceride; TC, total cholesterol; HDL, high-density lipoprotein cholesterol; LDL, low-density lipoprotein cholesterol.

**Table 2:** Baseline clinical characteristics in the cardiovascular group and the noncardiovascular group in men.

	Total (*n*=1194)	Non-CVD group (*n*=1089)	CVD group (*n*=105)	*t* value	*p* value
Age (years)	67.22 ± 12.11	66.75 ± 12.27	72.02 ± 8.95	−5.55	<0.001
BMI (kg/m^2^)	25.30 ± 3.51	25.25 ± 3.47	25.81 ± 3.88	−1.57	0.118
Diabetic duration (years)	10.1 (6.1, 15.3)	10.0 (6.0, 15.2)	12.8 (7.1, 16.8)	2.54^*∗*^ (*Z*)	0.011
Smoking (*n*, %)	386 (32.33)	358 (32.87)	28 (26.67)	1.69^#^ (x2)	0.194
WC (cm)	91.10 ± 8.81	90.90 ± 8.78	93.20 ± 8.90	−2.56	0.010
NC (cm)	38.20 ± 3.80	38.22 ± 3.73	38.02 ± 4.49	0.43	0.665
SBP (mmHg)	129.53 ± 14.50	129.26 ± 14.37	132.36 ± 15.59	−2.08	0.037
DBP (mmHg)	78.54 ± 9.06	78.54 ± 9.03	78.52 ± 9.35	0.02	0.984
FPG (mmol/L)	7.90 ± 2.69	7.89 ± 2.73	7.99 ± 2.27	−0.41	0.681
HbA1c (%)	7.39 ± 1.67	7.38 ± 1.69	7.57 ± 1.46	−1.10	0.270
TG (mmol/L)	1.80 ± 1.48	1.80 ± 1.51	1.74 ± 1.23	0.50	0.621
TC (mmol/L)	5.03 ± 1.20	5.02 ± 1.20	5.13 ± 1.18	−0.93	0.352
HDL (mmol/L)	1.24 ± 0.41	1.24 ± 0.42	1.19 ± 0.35	1.53	0.128
LDL (mmol/L)	2.94 ± 0.88	2.93 ± 0.89	3.00 ± 0.75	−0.82	0.416
Antihypertension medication (*n*, %)	669 (56.03)	597 (54.82)	72 (68.57)	7.35^#^ (x2)	0.007
Antiglycemic medication (*n*, %)	1073 (89.87)	971 (89.16)	102 (97.14)	6.69^#^ (x2)	0.010
Statin (*n*, %)	730 (61.14)	661 (60.7)	69 (65.71)	1.01^#^ (x2)	0.314

^*∗*^Rank sum test was used, expressed as median (range). ^#^Chi-square was used. CVD, cardiovascular disease; BMI, body mass index; WC, waist circumference; NC, neck circumference; SBP, systolic blood pressure; DBP, diastolic blood pressure; FPG, fasting plasma glucose; HbA1c, hemoglobin A1c; TG, triglyceride; TC, total cholesterol; HDL, high-density lipoprotein cholesterol; LDL, low-density lipoprotein cholesterol.

**Table 3:** Baseline clinical characteristics in the cardiovascular group and the noncardiovascular group in women.

	Total (*n*=1815)	Non-CVD group (*n*=1709)	CVD group (*n*=106)	*t* value	*p* value
Age (years)	67.88 ± 9.64	67.77 ± 9.72	69.57 ± 8.20	−2.16	0.033
BMI (kg/m^2^)	26.79 ± 57.28	26.86 ± 59.02	25.79 ± 3.50	0.73	0.466
Diabetic duration (years)	10.1 (6.8, 15.4)	10.0 (6.7, 15.3)	10.9 (8.0, 16.0)	1.75^*∗*^ (*Z*)	0.079
Smoking (*n*, %)	93 (5.13)	83 (4.86)	10 (9.43)	4.29^#^ (x2)	0.038
WC (cm)	87.72 ± 9.64	87.59 ± 9.54	89.82 ± 10.95	−2.05	0.043
NC (cm)	35.26 ± 3.40	35.21 ± 3.38	36.07 ± 3.55	−2.53	0.012
SBP (mmHg)	129.41 ± 14.67	129.05 ± 14.40	135.18 ± 17.52	−3.53	0.001
DBP (mmHg)	76.94 ± 8.90	77.00 ± 8.64	76.01 ± 12.40	0.81	0.422
FPG (mmol/L)	7.78 ± 2.48	7.77 ± 2.50	7.86 ± 2.30	−0.35	0.724
HbA1c (%)	7.32 ± 1.54	7.30 ± 1.54	7.60 ± 1.50	−1.89	0.059
TG (mmol/L)	1.88 ± 1.24	1.87 ± 1.22	2.11 ± 1.58	−1.48	0.142
TC (mmol/L)	5.35 ± 1.24	5.33 ± 1.24	5.60 ± 1.16	−2.07	0.038
HDL (mmol/L)	1.38 ± 0.50	1.37 ± 0.46	1.45 ± 0.89	−0.84	0.404
LDL (mmol/L)	3.15 ± 0.95	3.14 ± 0.94	3.33 ± 1.00	−1.95	0.052
Antihypertension medication (*n*, %)	1136 (62.59)	1056 (61.79)	80 (75.47)	7.98^#^ (x2)	0.005
Antiglycemic medication (*n*, %)	1660 (91.46)	1563 (91.46)	97 (91.51)	0.00^#^ (x2)	0.985
Statin (*n*, %)	1218 (67.11)	1146 (67.06)	72 (67.92)	0.03^#^ (x2)	0.854

^*∗*^Rank sum test was used, expressed as median (range). ^#^Chi-square was used. CVD, cardiovascular disease; BMI, body mass index; WC, waist circumference; NC, neck circumference; SBP, systolic blood pressure; DBP, diastolic blood pressure; FPG, fasting plasma glucose; HbA1c, hemoglobin A1c; TG, triglyceride; TC, total cholesterol; HDL, high-density lipoprotein cholesterol; LDL, low-density lipoprotein cholesterol.

**Table 4:** The hazard ratio and 95% confidence intervals of neck circumference and cardiovascular events in the Cox regression analysis.

	Hazard ratio	95% confidence interval	*p* value
Model 1			
Men	2.126	1.231–3.673	0.007
Women	2.043	1.244–3.356	0.005
Model 2	2.051	1.420–2.962	*p* < 0.001
Model 3	2.305	1.535–3.460	*p* < 0.001

Model 1: unadjusted. Model 2: adjusted age, smoking, and gender. Model 3: adjusted age, smoking, gender, SBP, HbA1c, LDL, diabetic duration, education attainments, the use of antihypertension medication, antiglycemic medication, and statin.

## Data Availability

All data generated and analysed during the current study are included in this article.

## References

[1] Haffner S. M., Lehto S., Rönnemaa T., Pyörälä K., Laakso M. (1998). Mortality from coronary heart disease in subjects with type 2 diabetes and in nondiabetic subjects with and without prior myocardial infarction. *New England Journal of Medicine*.

[2] Brun E., Nelson R. G., Bennett P. H. (2000). Diabetes duration and cause-specific mortality in the verona diabetes study. *Diabetes Care*.

[3] Mozaffarian D., Benjamin E. J., Go A. S. (2016). Heart disease and stroke statistics—2016 update: a report from the American Heart Association. *Circulation*.

[4] Selvin E., Parrinello C. M., Sacks D. B., Coresh J. (2014). Trends in prevalence and control of diabetes in the United States, 1988–1994 and 1999–2010. *Annals of Internal Medicine*.

[5] Preis S. R., Pencina M. J., Hwang S.-J. (2009). Trends in cardiovascular disease risk factors in individuals with and without diabetes mellitus in the Framingham heart study. *Circulation*.

[6] Luo Y., Ma X., Shen Y. (2017). Neck circumference as an effective measure for identifying cardio-metabolic syndrome: a comparison with waist circumference. *Endocrine*.

[7] Laakso M., Matilainen V., Keinänen-Kiukaanniemi S. (2002). Association of neck circumference with insulin resistance-related factors. *International Journal of Obesity*.

[8] Yang L., Samarasinghe Y. P., Kane P., Amiel S. A., Aylwin S. J. B. (2010). Visceral adiposity is closely correlated with neck circumference and represents a significant indicator of insulin resistance in WHO grade III obesity. *Clinical Endocrinology*.

[9] Liang J., Teng F., Li Y. (2013). Neck circumference and insulin resistance in Chinese adults: the cardiometabolic risk in Chinese (CRC) study. *Diabetes Care*.

[10] Onat A., Hergenç G., Yüksel H. (2009). Neck circumference as a measure of central obesity: associations with metabolic syndrome and obstructive sleep apnea syndrome beyond waist circumference. *Clinical Nutrition*.

[11] Ben-Noun L. L., Laor A. (2006). Relationship between changes in neck circumference and cardiovascular risk factors. *Experimental and Clinical Cardiology*.

[12] Kumar N. V., Ismail M. H., P. M., M. G., Tripathy M. (2014). Neck circumference and cardio- metabolic syndrome. *Journal of Clinical and Diagnostic Research*.

[13] Liang J., Wang Y., Li H., Liu X., Qiu Q., Qi L. (2014). Neck circumference and early stage atherosclerosis: the cardiometabolic risk in Chinese (CRC) study. *Cardiovascular Diabetology*.

[14] Preis S. R., Massaro J. M., Hoffmann U. (2010). Neck circumference as a novel measure of cardiometabolic risk: the Framingham heart study. *The Journal of Clinical Endocrinology & Metabolism*.

[15] Dai Y., Wan X., Li X., Jin E., Li X. (2016). Neck circumference and future cardiovascular events in a high-risk population—a prospective cohort study. *Lipids in Health and Disease*.

[16] Arjmand G., Shidfar F., Nojoomi M. M., Amirfarhangi A. (2015). Anthropometric indices and their relationship with coronary artery diseases. *Health Scope*.

[17] Yang G.-R., Yuan S.-Y., Fu H.-J. (2015). Influence of educational attainments on long term glucose control and morbid events in patients with type 2 diabetes receiving integrated care from 15 China urban communities: the Beijing community diabetes study 11. *Primary Care Diabetes*.

[18] Chinese Diabetes Society (2008). China guideline for type 2 diabetes (2007). *National Medical Journal of China*.

[19] Yang G.-r., Yuan S.-y., Fu H.-j. (2010). Neck circumference positively related with central obesity, overweight, and metabolic syndrome in Chinese subjects with type 2 diabetes: beijing community diabetes study 4. *Diabetes Care*.

[20] Sjöström C. D., Håkangård A. C., Lissner L., Sjöström L. (1995). Body compartment and subcutaneous adipose tissue distribution—risk factor patterns in obese subjects. *Obesity Research*.

[21] Ben-Noun L. L., Laor A. (2003). Relationship of neck circumference to cardiovascular risk factors. *Obesity Research*.

[22] Zhou J.-y., Ge H., Zhu M.-f. (2013). Neck circumference as an independent predictive contributor to cardio-metabolic syndrome. *Cardiovascular Diabetology*.

[23] Kaulgud R. S., Kaul A., Arun B. S., Vijayalaxmi P. B. (2017). Neck circumference and leg length as surrogate markers of coronary artery disease—simplifying cardiac risk stratification. *Journal of Clinical and Diagnostic Research*.

[24] Baena C. P., Lotufo P. A., Santos I. S. (2016). Neck circumference is associated with carotid intimal-media thickness but not with coronary artery calcium: results from the ELSA-Brasil. *Nutrition, Metabolism and Cardiovascular Diseases*.

